# Cholesterol nanoarchaeosomes for alendronate targeted delivery as an anti-endothelial dysfunction agent

**DOI:** 10.3762/bjnano.15.46

**Published:** 2024-05-13

**Authors:** Horacio Emanuel Jerez, Yamila Roxana Simioni, Kajal Ghosal, Maria Jose Morilla, Eder Lilia Romero

**Affiliations:** 1 Nanomedicine Research and Development Centre (NARD), Science and Technology Department, National University of Quilmes, Roque Sáenz Peña 352, Bernal, Buenos Aires, Argentina; 2 Department of Pharmaceutical Technology, Jadavpur University, 188, Raja Subodh Chandra Mallick Rd., Jadavpur, Kolkata 700032, West Bengal, India

**Keywords:** alendronate, archaeolipids, endothelial, inflammation

## Abstract

Sodium alendronate (ALN) is a very hydrosoluble and poorly permeable molecule used as an antiresorptive agent and with vascular anticalcifying capacity. Loaded into targeted nanovesicles, its anti-inflammatory activity may be amplified towards extra-osseous and noncalcified target cells, such as severely irritated vascular endothelium. Here cytotoxicity, mitochondrial membrane potential, ATP content, and membrane fluidity of human endothelial venous cells (HUVECs) were determined after endocytosis of ALN-loaded nanoarchaeosomes (nanoARC-Chol(ALN), made of polar lipids from *Halorubrum tebenquichense*: cholesterol 7:3 w/w, 166 ± 5 nm, 0.16 ± 0.02 PDI, −40.8 ± 5.4 mV potential, 84.7 ± 21 µg/mg ALN/total lipids, TL). The effect of nanoARC-Chol(ALN) was further assessed on severely inflamed HUVECs. To that aim, HUVECs were grown on a porous barrier on top of a basal compartment seeded either with macrophages or human foam cells. One lighter and one more pronounced inflammatory context was modelled by adding lipopolysaccharide (LPS) to the apical or the apical and basal compartments. The endocytosis of nanoARC-Chol(ALN), was observed to partly reduce the endothelial-mesenchymal transition of HUVECs. Besides, while 10 mg/mL dexamethasone, 7.6 mM free ALN and ALN-loaded liposomes failed, 50 μg/mL TL + 2.5 μg/mL ALN (i.e., nanoARC-Chol(ALN)) reduced the IL-6 and IL-8 levels by, respectively, 75% and 65% in the mild and by, respectively, 60% and 40% in the pronounced inflammation model. This is the first report showing that the endocytosis of nanoARC-Chol(ALN) by HUVECs magnifies the anti-inflammatory activity of ALN even under conditions of intense irritation, not only surpassing that of free ALN but also that of dexamethasone.

## Introduction

Many acute lethal events and chronic inflammatory diseases are mediated by initial or consequential damage to the vascular endothelium. Endothelial stress manifests as changes in vascular tone, increased permeability, thrombotic events, and exposure to circulating leukocyte receptors. Different pharmacological agents can reduce endothelial dysfunction [1], but they have no effect on acute inflammations or edema such as those occurring in sepsis, a potentially lethal condition that induces profound hemodynamic alterations [2].

Bisphosphonates are synthetic, nonhydrolyzable analogs of inorganic pyrophosphate [3] that are clinically employed to remove osteoclasts in the treatment of osteoporosis and tumors and reduce bone mineralization [4]. Interestingly, besides targeting areas of active bone remodeling and resorption [5] and osteoclasts, nitrogenous bisphosphonates have been reported to reduce vascular calcification, through direct or indirect interaction with the endothelium [1,6]. Alendronate sodium (ALN) (CAS 121268-17-5, 4-amino-1-hydroxybutylidine-1,1-bisphosphonic acid) is a nitrogenous bisphosphonate with high affinity for the bone matrix of hydroxyapatite, chelating capacity of divalent cations, and anti-osteoclast activity, widely used in clinical settings as an anti-resorptive agent in osteoporosis [7–9]. ALN is known to reduce arterial calcification at doses comparable to those that inhibit bone resorption [10] and reported to exhibit a proangiogenic action on stressed endothelial cells, enhancing vascular endothelial growth factor (VEGF) synthesis and inducing the formation of capillary-like tubes in a VEGF-dependent manner [11]. Recent studies have also shown that ALN has a direct anti-inflammatory effect on endothelial cells. It reduces LPS-induced activation in terms of expression of cell adhesion molecules to leukocytes and increases the production of nitric oxide, reducing platelet activation [12].

According to the Biopharmaceutical Classification System, ALN is a class-III molecule (high solubility and low permeability due to a polar hydrophilic nature) [13]. Its use as a vascular anti-inflammatory agent is limited by its low bioavailability, which after oral administration is minimal (about 0.7%) and highly variable [14–16]. After a single oral dose of ALN (70 mg tablet), a peak plasma concentration of 33.10 ± 14.32 ng/mL (≈0.15 μM) of ALN is attained after 1.00 ± 0.16 h [17]. This plasma concentration is about 170 times lower than the average of 1–50 μM ALN reported for the in vitro anti-inflammatory activity of ALN on endothelial cells. This is important because the access of ALN in therapeutically realistic concentrations to the inflamed endothelium is limited to microenvironments exhibiting the abnormal presence of hydroxyapatite in the vessel structure. ALN associates with great affinity to this mineral matrix, typical of cardiovascular pathologies, and is desorbed from it in an acid medium, locally reaching micro- and even millimolar concentrations [18].

Delivering high doses of ALN to extra-osseous targets, such as blood cells or inflamed vascular walls, in order to profit from its anti-inflammatory properties is a pharmacological challenge that could be addressed by formulating ALN in nanomedicines. Properly designed, intravenously administered nanomedicines allow one to control pharmacokinetics, biodistribution, and pharmacodynamics of loaded active ingredients [19]. Inflamed endothelia present variable degrees of increased permeability, offering an anatomic-pathological context that favors extravasation and, therefore, the passive targeting of nanoparticulate material towards cells of the diseased vasculature [20]. Moreover, in recent years, the development of targeted nanomedicines for therapeutic, diagnostic, or theragnostic applications to pathological macrophages and endothelia is of major pharmaceutical interest [21]. The vascular endothelium can be actively targeted with nanomedicines of high structural sophistication [22–23], which are, however, difficult to fit within the increasingly pursued “quality by design” criteria [24]. The development of structurally simple formulations that facilitate their scaling and further characterization is, therefore, a critical task [25]. Also, targeting nanomedicines to circulating cells and vascular walls is difficult since it should occur with sufficient effectivity under dynamic conditions [26]. However, simple in vitro experimental settings employing static conditions could anticipate both potential toxicity and therapeutic effects.

In this context, new natural biomaterials such as archaeolipids are being explored with growing interest in the drug delivery field [27–28]. Nanoarchaeosomes (nanoARC) prepared with lipids extracted from *H. tebenquichense*, for example, are naturally targeted to scavenger receptor A I/II (SRAI/II) expressed by phagocytic cells and certain endothelial cells and outperform liposomes in structural simplicity and resistance to mechanical stress. Recently, we have reported the structural characterization and effect on J774A.1 murine macrophages of ALN loaded in nanoarchaeosomes, that is, nanoARC(ALN) and nanoARC-Chol(ALN). Remarkably, these formulations do not seek to modify the solubility of ALN, but to trap it in naturally targeted nanomedicines to grant its massive intracellular delivery upon endocytosis. The endocytosis of nanoARC(ALN) by J774A.1 macrophages had a pro-apoptotic effect, while nanoARC-Chol(ALN) and void nanoARC-Chol presented intense anti-inflammatory activity; free ALN at identical micromolar concentration, in contrast, had no anti-inflammatory effect [29].

Although macrophages are cells specialized in phagocytizing, and endothelial cells make up the structure of the vasculature and maintain a complex state of vigilance to blood signals, similarities between both cell types have lately been described [30–32]. Endothelial cells express a diversity of innate immune receptors including Toll-like receptors (TLRs) and NOD-like receptors (NLRs), which activate intracellular inflammatory pathways mediated by nuclear factor kappa B (NF-kB) and the mitogen-activated protein kinases (MAPKs). Both share additional important similarities such as the ability to display autophagy and to phagocytose particulate material [33].

The endocytosis of ALN-loaded nanoarchaeosomes from *H. tebenquichense* by HUVECs intensely inflamed by LPS and its effect are presented here for the first time. To this end, we used an experimental setting consisting of HUVECs growing on a porous barrier on top of a basal compartment seeded either with macrophages or human foam cells (FCs). A lighter or more pronounced inflammatory context was modeled by adding LPS to the apical or the apical and basal compartments, respectively. Our main findings were that the endocytosis of nanoARC-Chol(ALN) by HUVECs reduced the endothelial-mesenchymal transition induced by LPS; also, while dexamethasone, micromolar-free ALN, and ALN-loaded HSPC-Chol liposomes failed, nanoARC-Chol(ALN) strongly reduced the production of IL-6 and IL-8 by HUVECs even in the presence of TNF-α.

## Results

### Characterization of ALN-loaded nanovesicles

**Figure 1:**
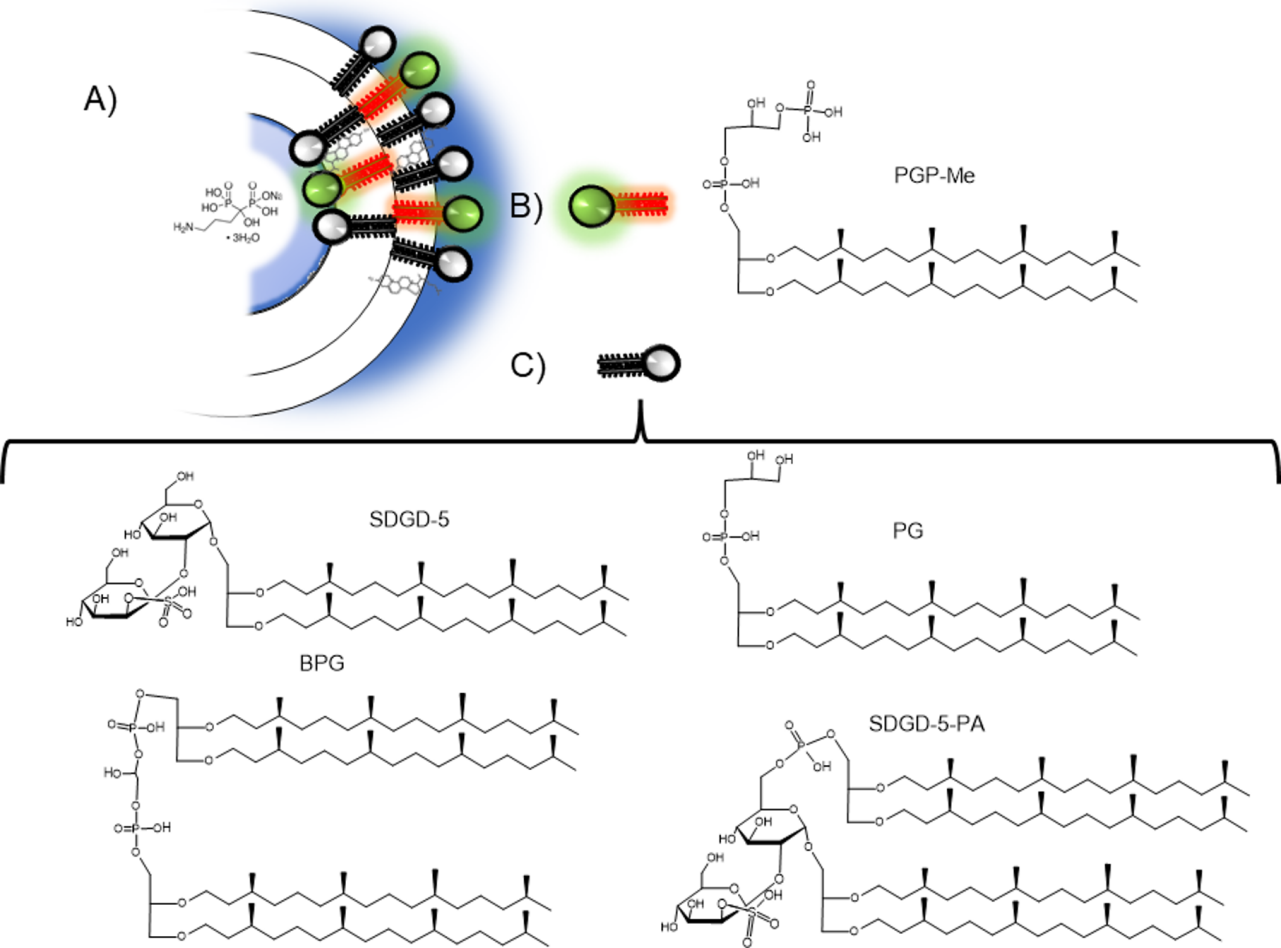
(A) Representative image of alendronate loaded in a cholesterol-containing nanoarchaeosome (nanoARC-Chol (ALN)). (B) Structure of phosphatidylglycerol phosphate methyl ester (PGP-Me). (C) Structure of the four polar archaeolipids composing the total polar lipids from *H. tebenquichense*.

The structural features of ALN-loaded nanovesicles are shown in Table 1. As reported previously, nanoarchaeosome bilayers contain archaeolipids displaying methyl groups perpendicular to their longitudinal axis (Figure 1) for trapping small molecules either into the bilayer or their aqueous space more efficiently than liposomes [34–35]. In this case, nanoarchaeosomes incorporated ALN to a higher extent than HSPC-Chol liposomes [29], as shown by their ≈1.4 times higher ALN/lipid ratio.

**Table 1:** Structural features of nanovesicles.^a^

Formulation	Size (nm ± SD)	PDI	ζ Potential (mV ± SD)	TL (mg/mL ± SD)	ALN (mg/mL ± SD)	ALN/TL (µg/mg ± SD)

nanoARC	161 ± 12	0.16 ± 0.03	−29.0 ± 2.1	6.7 ± 1.5	—	—
nanoARC-Chol	173 ± 4.3	0.16 ± 0.02	−31.6 ± 2.1	6.5 ± 1.3	–	—
HSPC-Chol	296 ± 42	0.42 ± 0.11	−4.9 ± 1.4	3.3 ± 0.4	—	—
nanoARC(ALN)	163 ± 4.8	0.16 ± 0.02	−37.1 ± 5.1	4.6 ± 0.9	0.46 ± 0.15	118 ± 67
nanoARC-Chol(ALN)	166 ± 5.2	0.17 ± 0.03	−40.8 ± 5.4	4.3 ± 0.6	0.40 ± 0.12	84.7 ± 21
HSPC-Chol(ALN)	246 ± 42	0.25 ± 0.10	−4.7 ± 0.8	4.1 ± 0.9	0.22 ± 0.09	59.2 ± 31

^a^Data are expressed as mean ± standard deviation from six independent batches. ALN: alendronate; HSPC: hydrogenated soy phosphatidylcholine; PDI: polydispersity index; SD: standard deviation; TL: total lipids.

### Cytotoxicity on HUVECs, human macrophages, and monocytes

**Figure 2:**
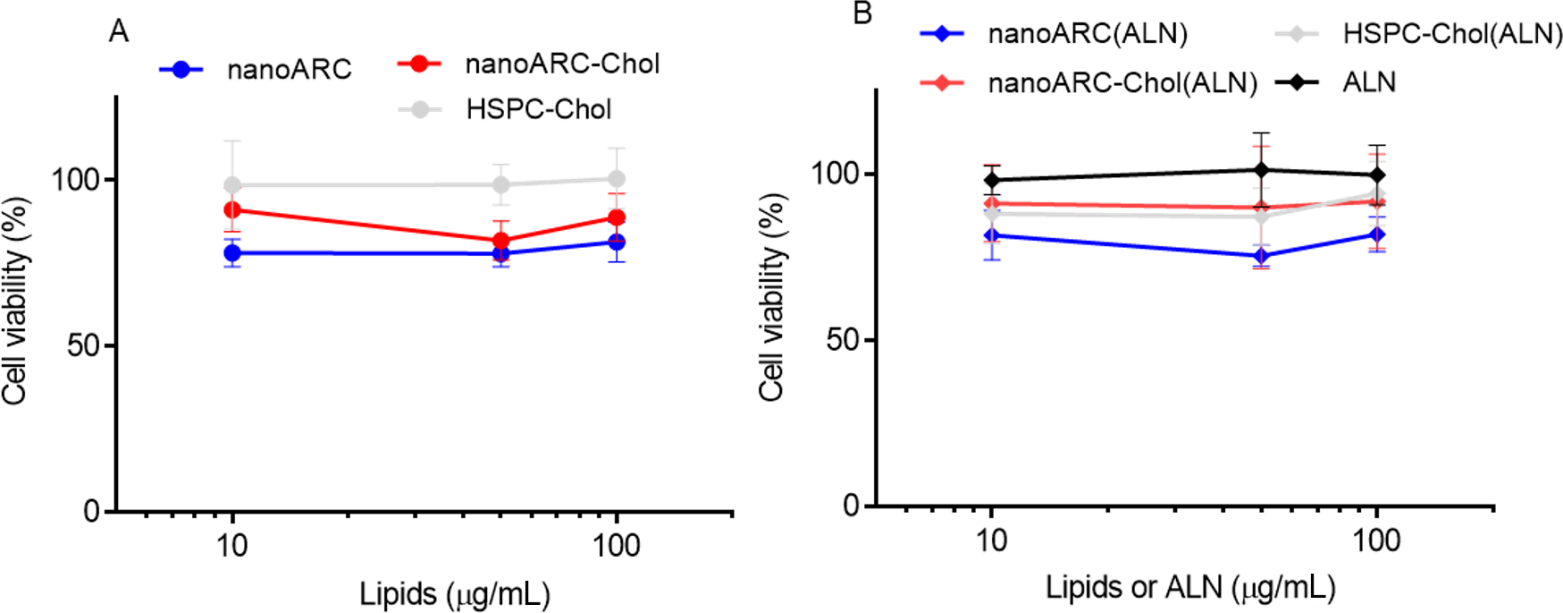
HUVEC viability upon nanovesicle uptake. Viability upon 24 h of incubation with (A) void nanovesicles and (B) ALN-loaded nanovesicles. Data are expressed as mean ± SD (*n* = 3).

Despite void nanoARC seemed to slightly reduce HUVEC viability by ≈20%, and nanoARC-Chol by 10%, whereas HSPC-Chol liposomes caused no reduction in cytotoxicity (Figure 2A), no statistically significant differences were found between the formulations and the control. The cytotoxicity was independent of the presence of ALN and lipid concentration in a range between 10 and 100 μg TL/mL (Figure 2B).

**Figure 3:**
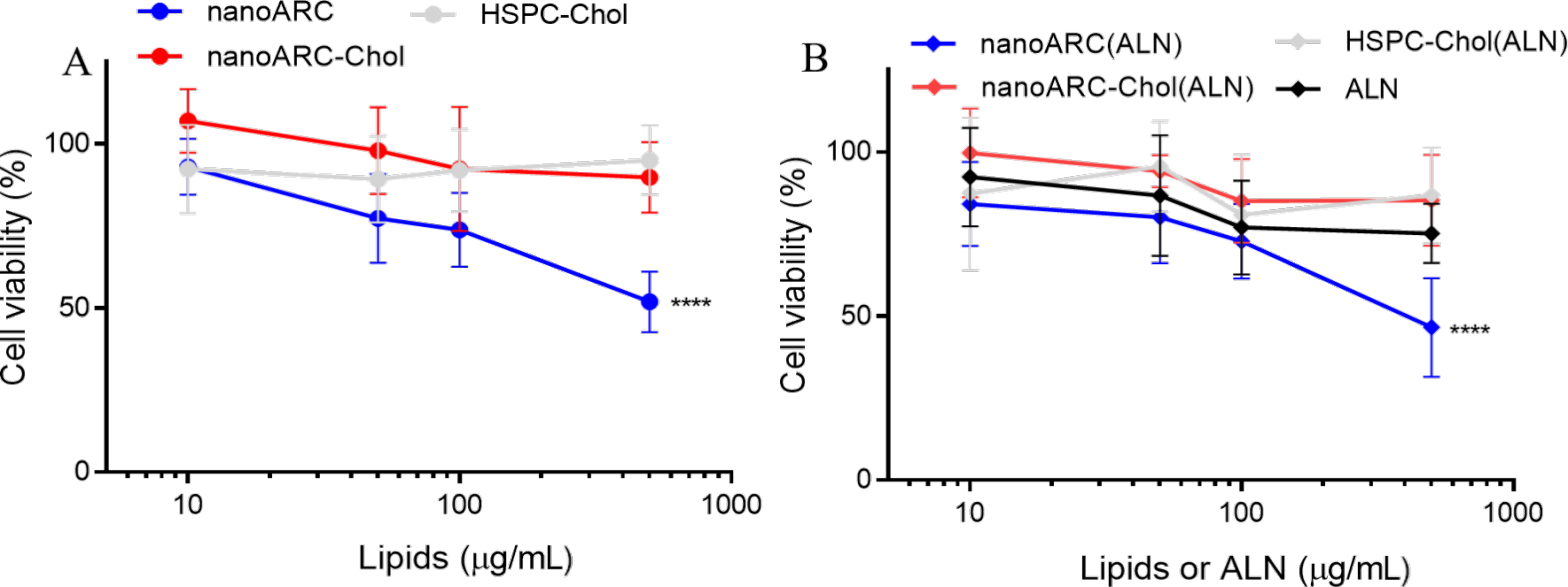
Viability of THP-1 macrophages upon 24 h of incubation with (A) void nanovesicles and (B) ALN- loaded nanovesicles. Data are expressed as mean ± SD (*n* = 3).

THP-1-derived macrophages were much less susceptible to nanoarchaeosomes than J774A.1 cells [29]. The IC50 for nanoARC(ALN) was ≈500 μg TL/mL (vs 100 μg TL/mL in J774A.1), and the IC50 for nanoARC-Chol(ALN) was ≫500 μg TL/mL (vs 500 μg TL/mL in J774A.1) (Figure 3).

**Figure 4:**
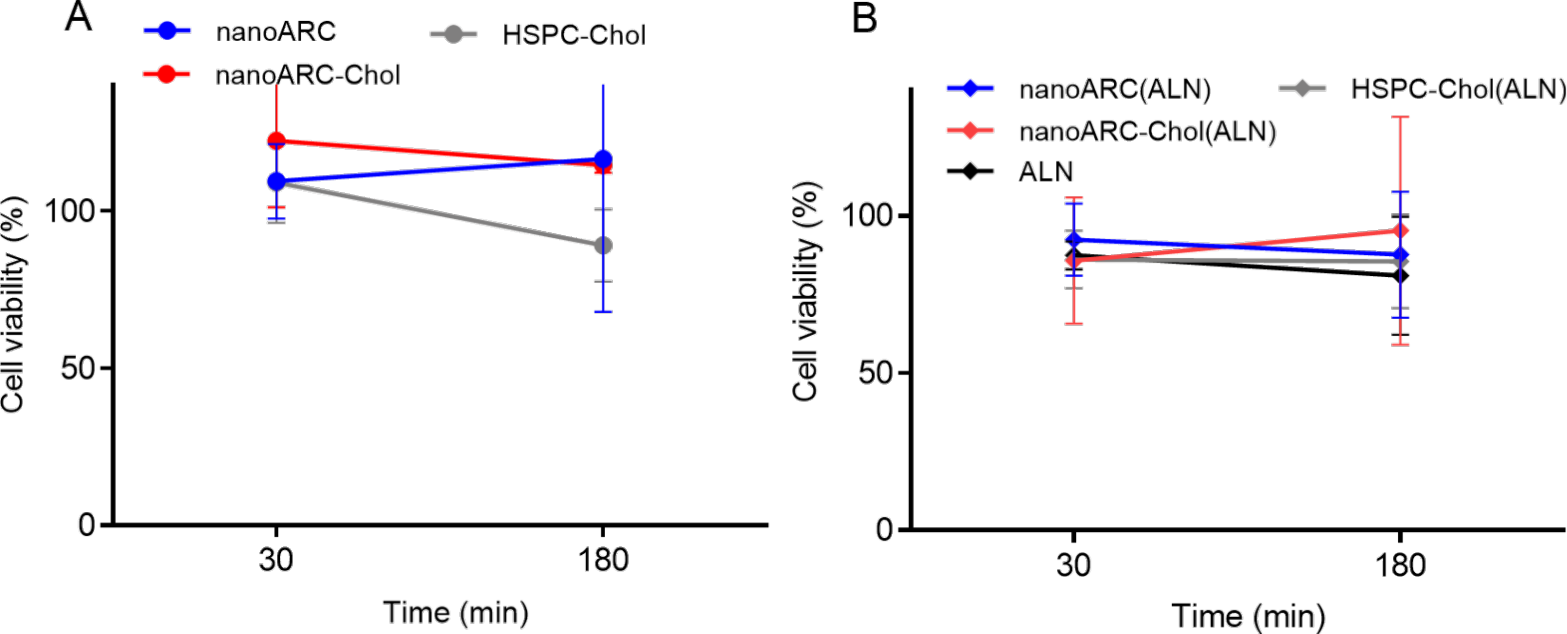
Viability of THP-1 monocytes upon 30 and 180 min of incubation with 500 μg TL/mL nanovesicles with (A) void nanovesicles and (B) ALN-loaded nanovesicles. Data are expressed as mean ± SD (*n* = 3).

The formulations were also not cytotoxic to circulating human monocytes between 100 and 500 μg/mL upon 30–180 min of incubation (Figure 4). No statistically significant differences were found between the formulations and the control.

**Figure 5:**
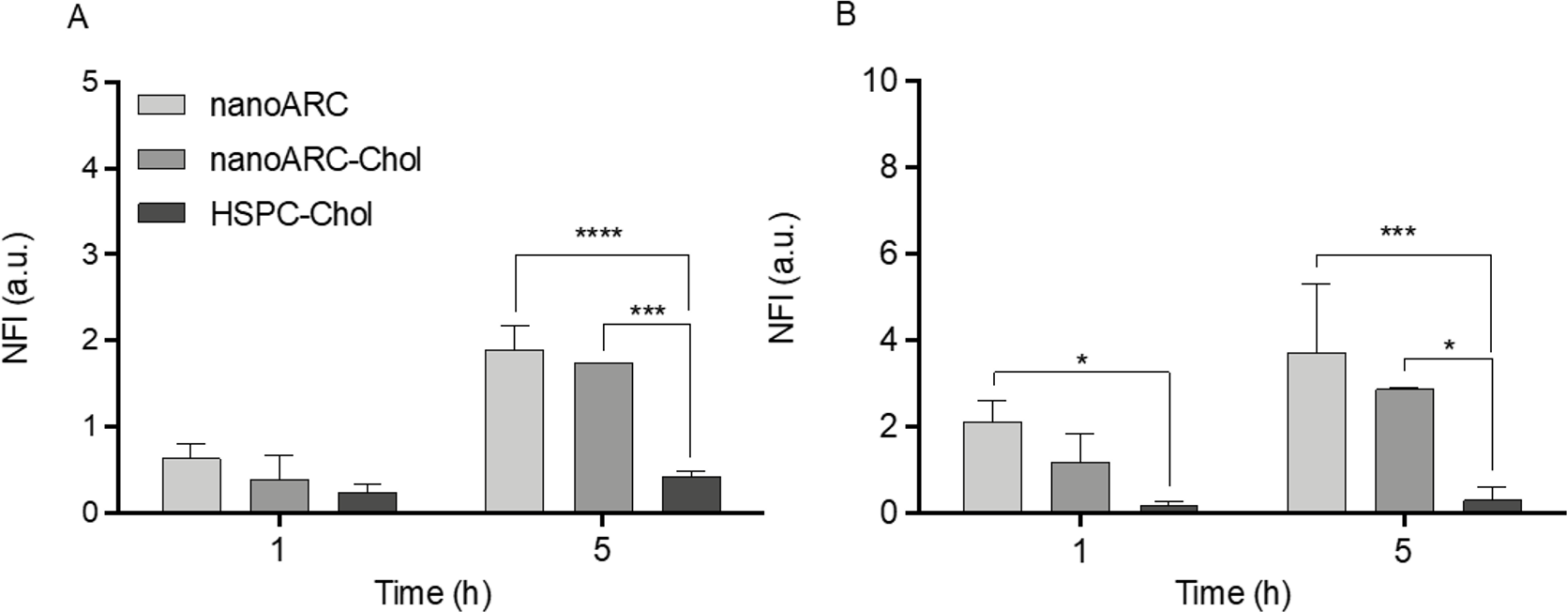
Uptake of nanovesicles by (A) THP-1 macrophages and (B) THP-1 monocytes. Data are expressed as mean ± SD (*n* = 3). NFI: normalized fluorescence intensity.

We observed, however, that the void formulations nanoARC and nanoARC-Chol were internalized by human macrophages and monocytes to a significantly higher extent than HSPC-Chol liposomes (Figure 5), as previously reported about J774A.1 macrophages [29]. Recently, we also reported the increased internalization of nanoarchaeosomes compared to HSPC-Chol liposomes by HUVECs [36]. As discussed below, the massive internalization of nanoarchaeosomes is due to their high content of PGP-Me, a ligand of SRA-I/II.

### Effect of endocytosis on HUVECs and THP-1 macrophages

**Figure 6:**
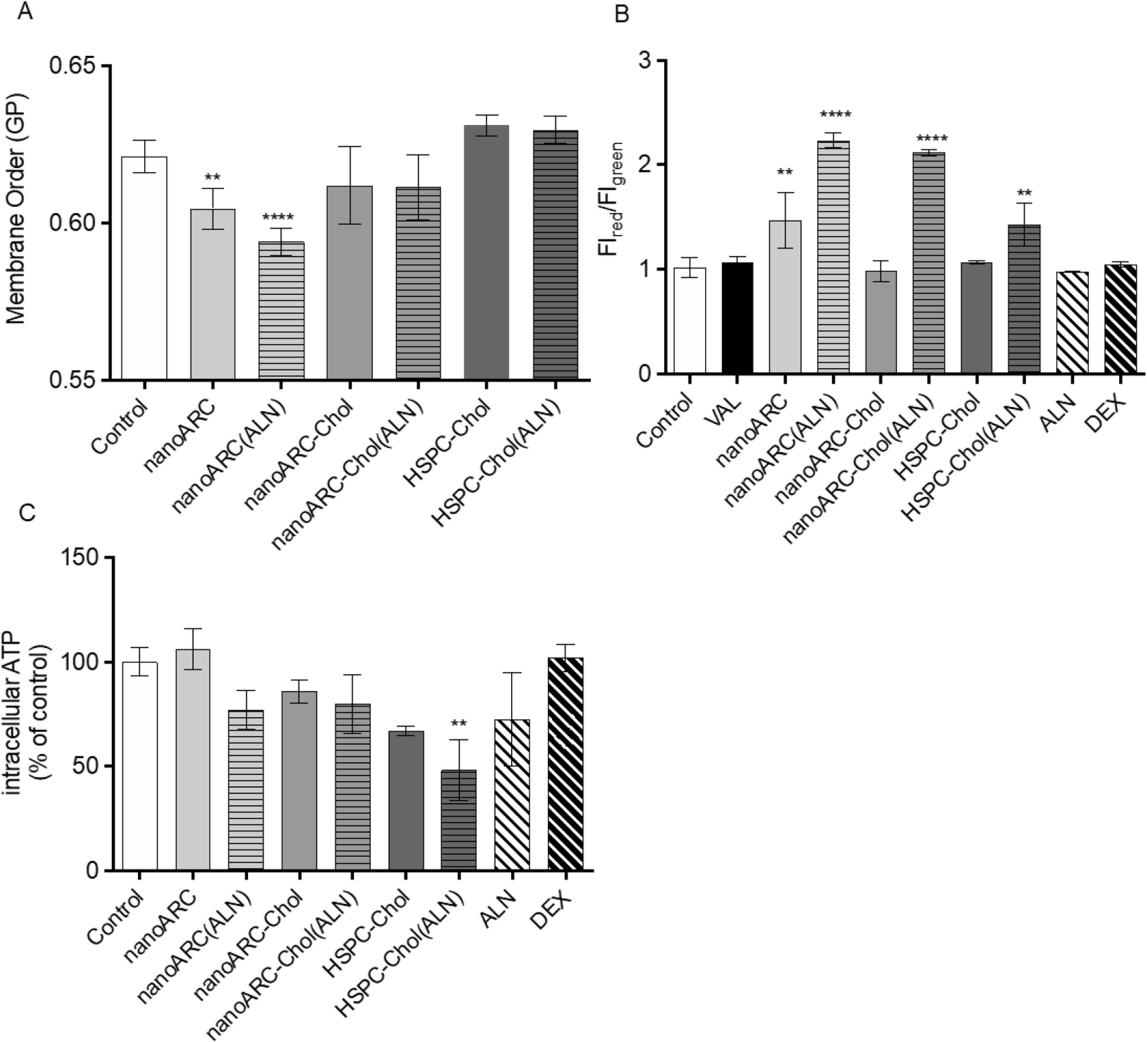
Effects of nanovesicle uptake by HUVECs on (A) plasma membrane order, (B) mitochondrial membrane potential, and (C) intracellular ATP. VAL: valinomycin. Data are expressed as mean ± SD (*n* = 3). Asterisks indicate significant differences to the control (medium).

The following effects were induced in HUVECs after the endocytosis of ALN-loaded nanovesicles: (i) nanoARC and nanoARC(ALN) disordered the plasma membrane of HUVECs, whereas nanoARC-Chol did not (Figure 6A). The results were consistent with membrane disorganization observed in halophilic archaeolipids [37–38] and the organizing role of cholesterol in archaeolipid bilayers [39]. The alteration in membrane order suggests that, after endocytosis, archaeolipids integrate into the plasma membrane of HUVECs. The endocytosis of ALN did not perturb the membrane organization. (ii) All formulations containing ALN caused mitochondrial membrane hyperpolarization (nanoARC(ALN) = nanoARC-Chol(ALN) > HSPC-Chol(ALN)), the same as void nanoARC (Figure 6B). (iii) Only HSPC-Chol(ALN) caused a significant reduction in ATP content (Figure 6C).

After ALN-loaded nanovesicles were endocytosed by HUVECs, no increase in nitric oxide (NO) production was registered under any of the following conditions: (i) 50 µg lipids/mL nanovesicles and 2.5 µg free ALN/mL for 24 h, (ii) 100 μg lipids/mL nanovesicles and 5 µg free ALN/mL for 15, 30, and 60 min, and (iii) 50 μg lipids/mL nanovesicles and 1, 5, 10, and 50 µM free ALN for 15, 30, and 60 min (data not shown).

**Figure 7:**
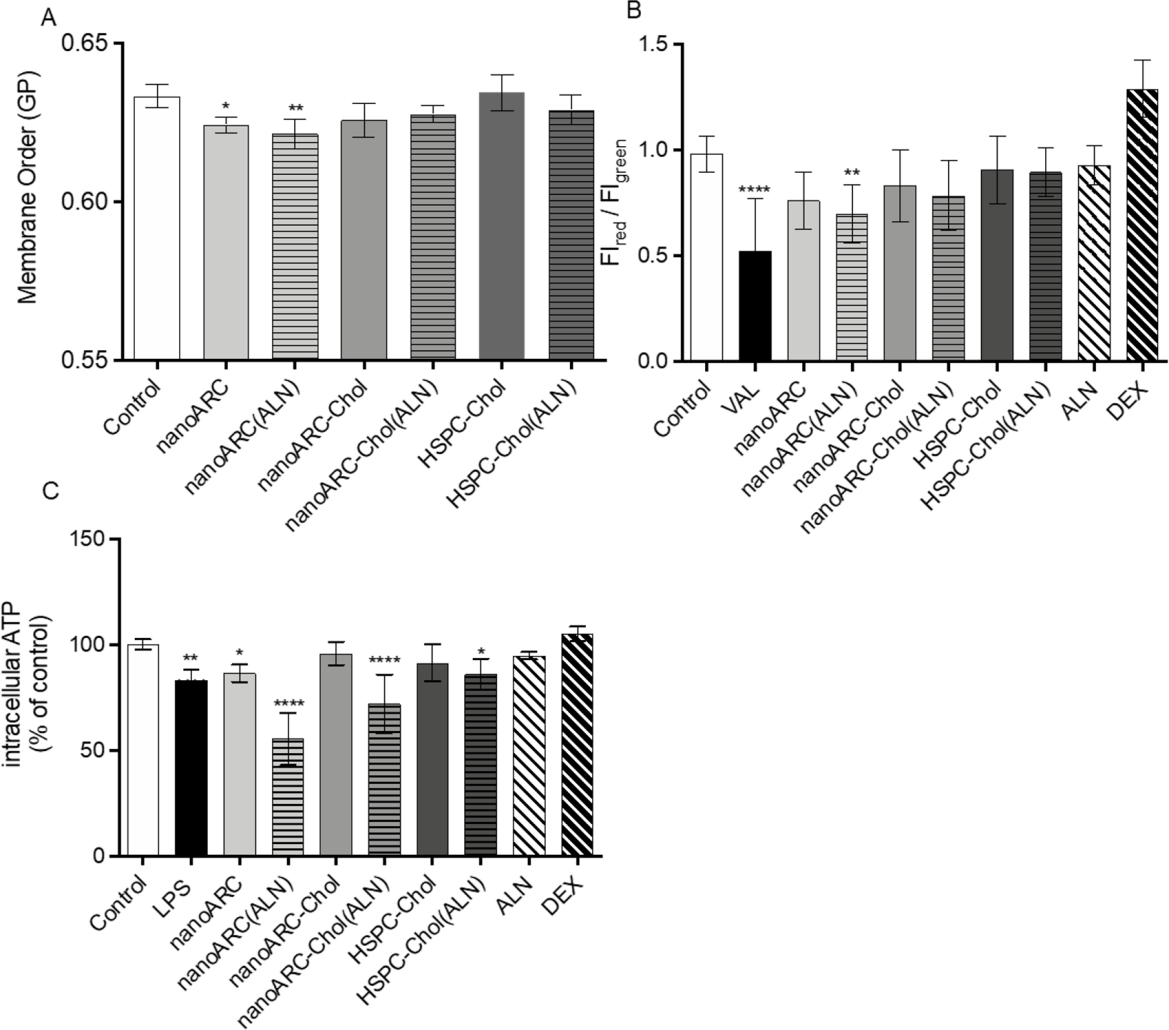
Effects of nanovesicle uptake by THP-1 macrophages on (A) plasma membrane order, (B) mitochondrial membrane potential, and (C) intracellular ATP. Data are expressed as mean ± SD (*n* = 3). Asterisks indicate significant differences against control (medium).

**Figure 8:**
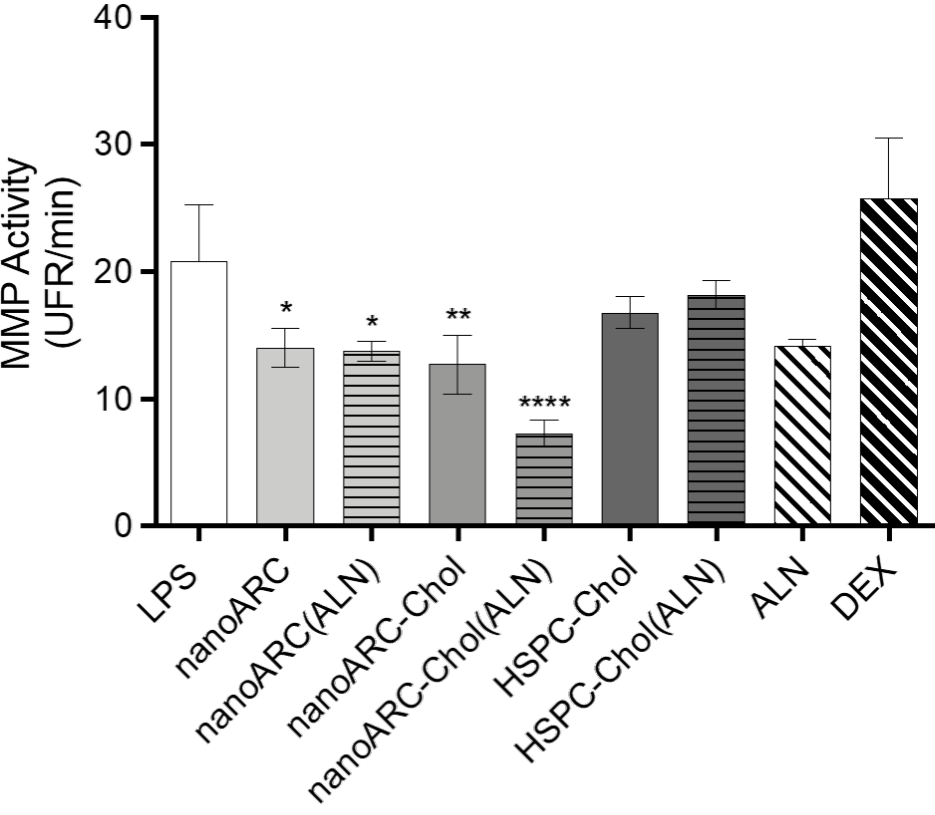
Effect of nanovesicle uptake on matrix metalloproteinases secreted by THP-1 macrophages. Data are expressed as mean ± SD (*n* = 3).

In human macrophages instead, the endocytosis of ALN-loaded nanovesicles induced a series of non-lethal changes that differed from those in HUVECs: (i) Again, nanoARC and nanoARC(ALN) disordered the plasma membrane of THP-1 macrophages, while nanoARC-Chol did not, and the presence of ALN did not affect its organization (Figure 7A). (ii) Different from HUVECs, the formulations did not cause mitochondria membrane potential hyperpolarization; only nanoARC(ALN) caused a slight but significant hypopolarization (Figure 7B). (iii) The ATP levels were sensitive to the formulations. nanoARC(ALN) decreased the intracellular levels of ATP by ≈50%, followed by nanoARC-Chol(ALN) with ≈25% (nanoARC ≅ LPS ≅ HSPC-Chol(ALN) (≈10%)), while the ATP levels remained unchanged after endocytosis of nanoARC-Chol, HSPC-Chol, ALN, and DEX (Figure 7C). All nanoarchaeosomes, void or loaded with ALN, had an anti-matrix metalloproteinase (MMP) effect. Free ALN caused a statistically not significant decrease of MMP activity. However, the endocytosis of 50 μg TL/mL nanoARC-Chol(ALN) yielded the highest anti-MMP2 activity (Figure 8).

A high proportion of archaeolipids from *H. tebenquichense* nanoarchaeosomes is PGP-Me, a polar double negatively charged archaeolipid, which is a ligand of SRAI/II (a trimeric transmembrane glycoprotein that mediates the extensive internalization of polyanionic ligands) [40]. SRAI/II is expressed by J774A.1 cells in intermediate amounts [41]. The expression of SRAI/II by THP-1 monocytes/macrophages, instead, is low [42–43] and is reduced by IL-6, making THP-1 cells more sensitive to the effect of LPS [44]. The expression of SRAI/II is also correlated with the state of activation of THP-1 macrophages; it is reduced in the pro-inflammatory phenotype M1. In contrast, J774A.1 cells express a phenotype close to M0 with greater expression of SRAI/II [41]. Therefore, the effect of nanoarchaeosomes may differ depending on, for instance, the cell genotype because of differences in phospholipid processing [45] and on the various factors that modulate the expression of SRAI/II. We observed here that nanoARC(ALN) was much less cytotoxic for THP-1 macrophages than for J774A1 cells, probably, because the expression of SRA1 in THP-1 macrophages (and subsequent internalization) is reported to be lower than in J774A1 macrophages [43].

### Co-cultures

#### Mild inflammation model

The previous crosstalk induced relatively low levels of IL-6 and IL-8 on the apical side with HUVECs (Supporting Information File 1, Figure S1). Then, in response to LPS, the levels of IL-6 and IL-8 raised to ≈600 pg/mL and ≈5000 pg/mL, respectively; no TNF-α was detected, a result consistent with previous reports [46]. Both IL-6 and IL-8 produced by HUVECs diffused downwards.

In the basal compartment, the IL-8 level rose from ca. 2000 to 5500 pg/mL in response to apical LPS. Macrophages, in addition to expressing prominent levels of IL-8 receptors [47–48] are the main inducers of IL-8 in the immune system*.* IL-8 induces in macrophages the production of IL-6, IL-1β but not of TNF-α [49]. The IL-6 level in the basal compartment, after adding LPS to the apical side, oscillated around 500 pg/mL*.* This IL-6 concentration should result from what diffused from the apical side plus what was induced in macrophages after IL-8 stimulation*.* In macrophages, IL-6 reinforces the pre-existing phenotype [50] (in this case, the inflammatory phenotype).

**Figure 9:**
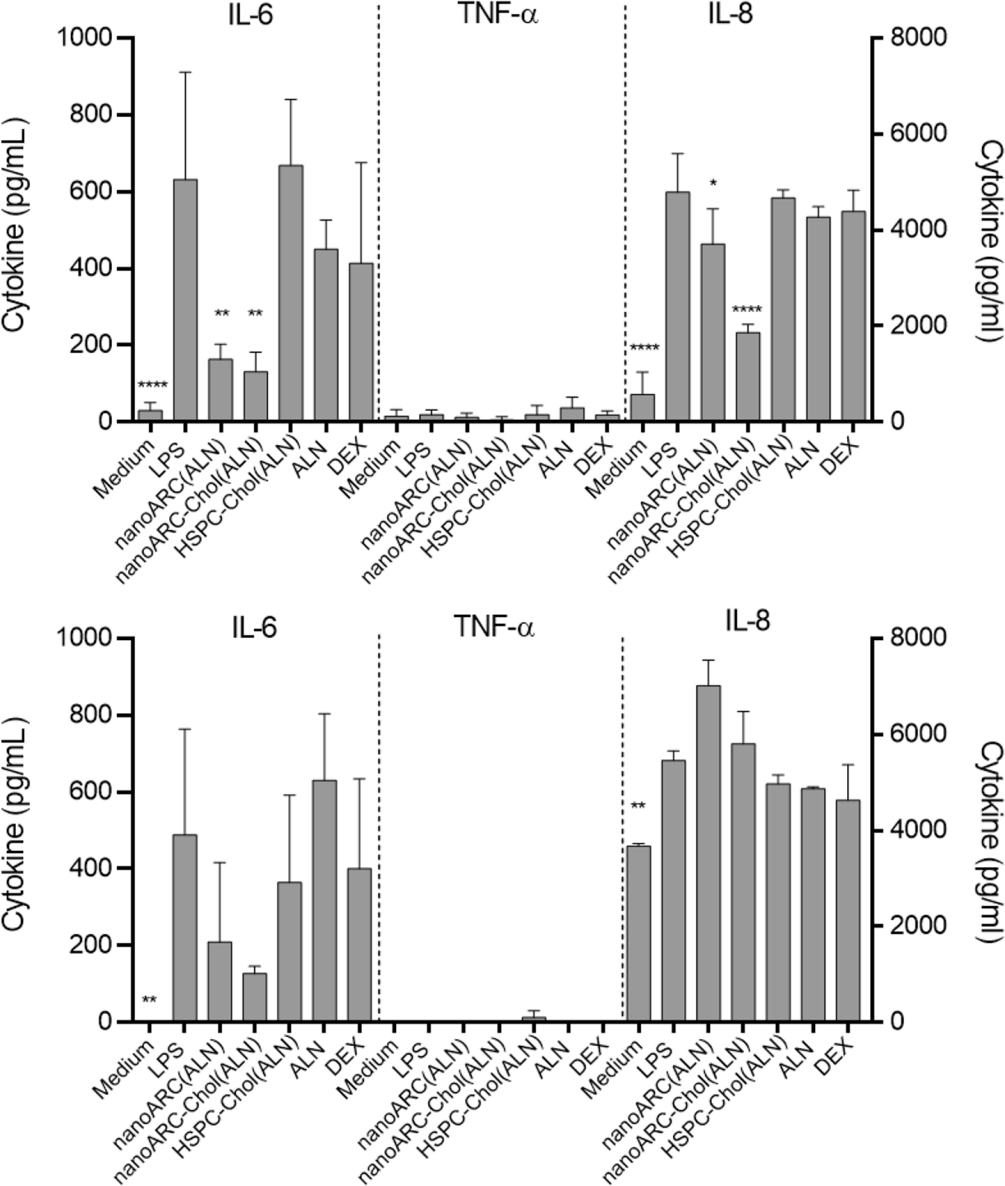
Effect of nanovesicle uptake on cytokines released in the mild inflammation model: (A) apical (HUVEC) and (B) basolateral (THP-1 macrophages). Values of IL-6 and TNF-α are shown on the left *Y* axis, and values of IL-8 are shown on the right *Y* axis. Data are expressed as mean ± SD (*n* = 3). Asterisks indicate significant differences against LPS.

**Figure 10:**
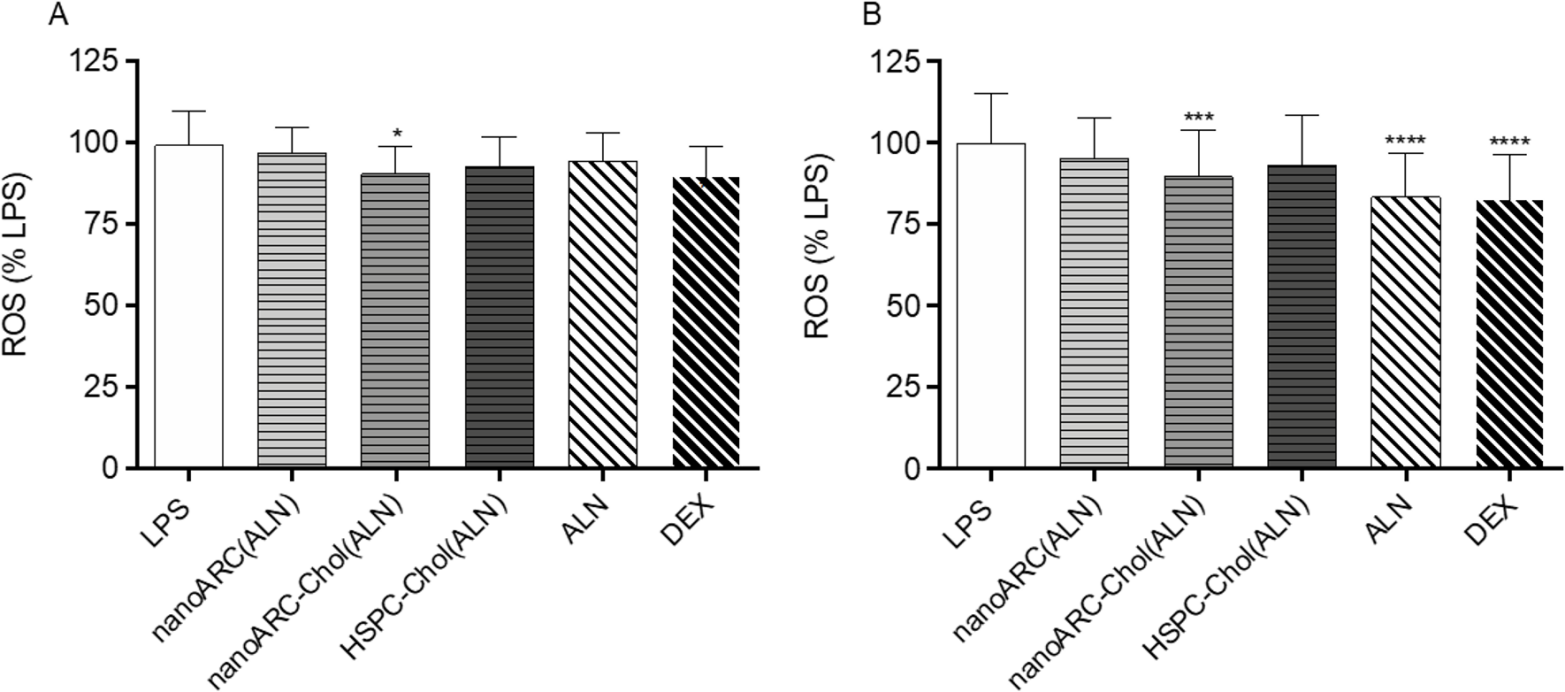
ROSs in (A) THP-1 macrophages and (B) FCs. Data are expressed as mean ± SD (*n* = 3).

We observed that ALN-loaded nanoarchaeosomes had a profound anti-inflammatory effect. nanoARC(ALN) and nanoARC-Chol(ALN) decreased the apical levels of IL-8 by ≈25% and 65%, respectively, and of IL-6 by 65% and 75%, respectively. In the basal compartment, both formulations reduced the level of IL-6 by 60% and 75%, respectively, while the level of IL-8 remained unchanged. Neither HSPC-Chol(ALN) endocytosis nor incubation with ALN or dexamethasone reduced IL-6 or IL-8 levels in either compartment (Figure 9). nanoARC-Chol(ALN) caused a slight decrease in reactive oxygen species (ROSs) in macrophages (Figure 10A).

#### Pronounced inflammation model

**Figure 11:**
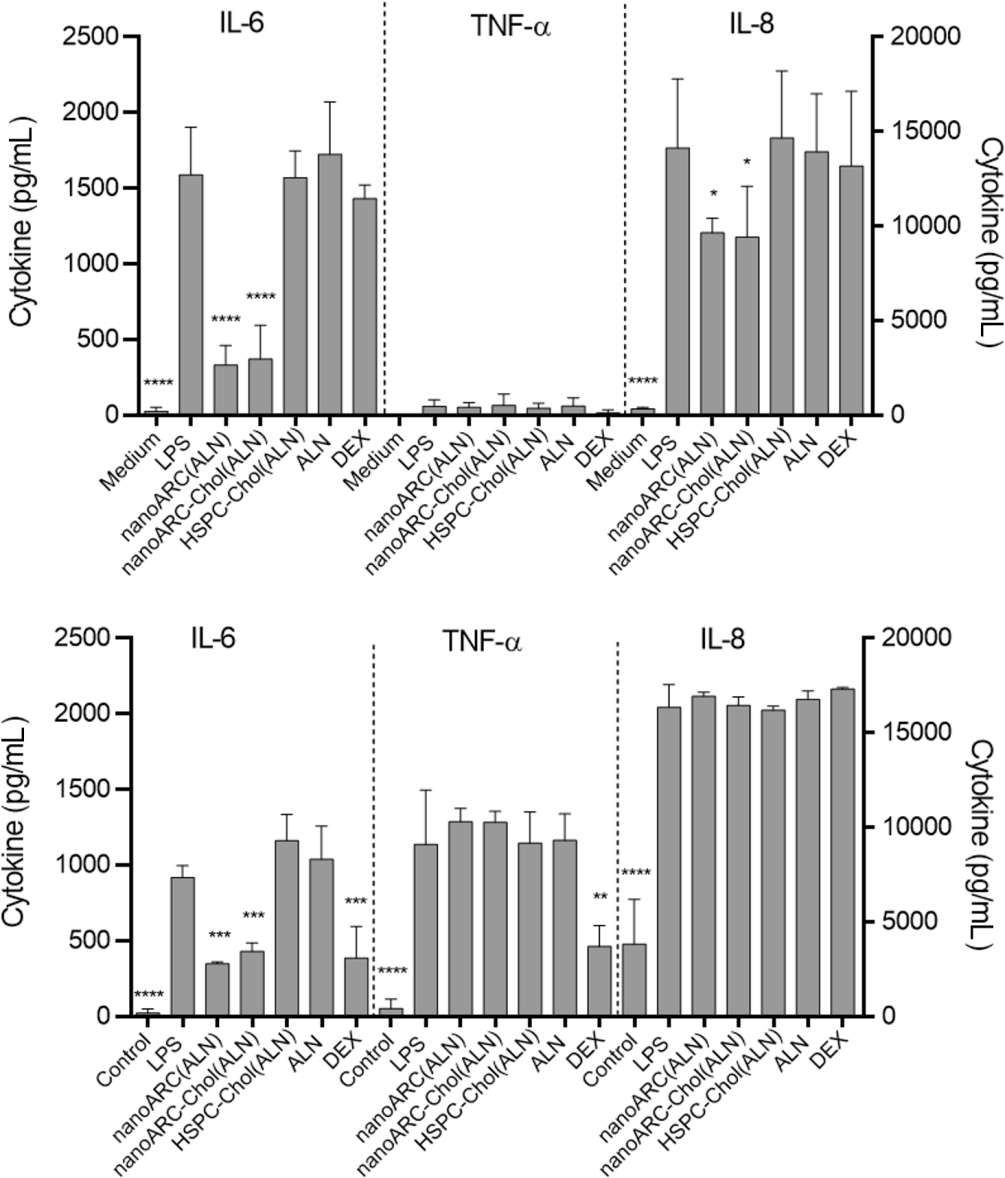
Effect of nanovesicle uptake on cytokines released in the pronounced inflammation model: (A) apical (HUVECs) and (B) basolateral (FCs). Values of IL-6 and TNF-α are shown on the left *Y* axis, and values of IL-8 are shown on the right *Y* axis. The levels of IL-6 and IL-8 in the basal compartment would be the sum of the amount produced by FCs plus what diffused from HUVEC*.* Data are expressed as mean ± SD (*n* = 3). Asterisks indicate significant differences against LPS.

The previous crosstalk induced mild levels of IL-6 and IL-8 on the apical side with HUVECs (Supporting Information File 1, Figure S2), suggesting that FCs were weakly pro-inflammatory, as described to happen when artificially induced [51]. Then, in response to LPS, IL-6 and IL-8 levels rose to 1600 and 14000 pg/mL, respectively. In the basal compartment the levels of IL-6 and IL-8 were 900 and 16000 pg/mL, respectively; 1200 pg/mL TNF-α were also induced. HUVECs on the apical side were exposed to the proliferative and increased vascular permeability stimulus of IL-8 and IL-6 and induced to express adhesion molecules and cytokine production by IL-6 and TNF-α [52] secreted in the basal compartment. In this more pronounced inflammatory context, we again observed that only nanoARC(ALN) and nanoARC-Chol(ALN) reduced the apical levels of IL-8 by ≈30% and of IL-6 by ≈80%. Neither HSPC-Chol(ALN) endocytosis nor ALN or dexamethasone reduced the apical levels of IL-6 or IL-8. In the basal compartment, IL-6 was reduced by 70% by both formulations, while the level of IL-8 remained unchanged. Dexamethasone reduced ≈70% and ≈40% of the IL-6 and TNF-α levels, respectively (Figure 11). Apical nanoARC-Chol(ALN), dexamethasone, and ALN decreased ROSs in human FCs (Figure 10B). Void formulations did not exhibit anti-inflammatory effects in any of the inflammation models (data not shown).

**Figure 12:**
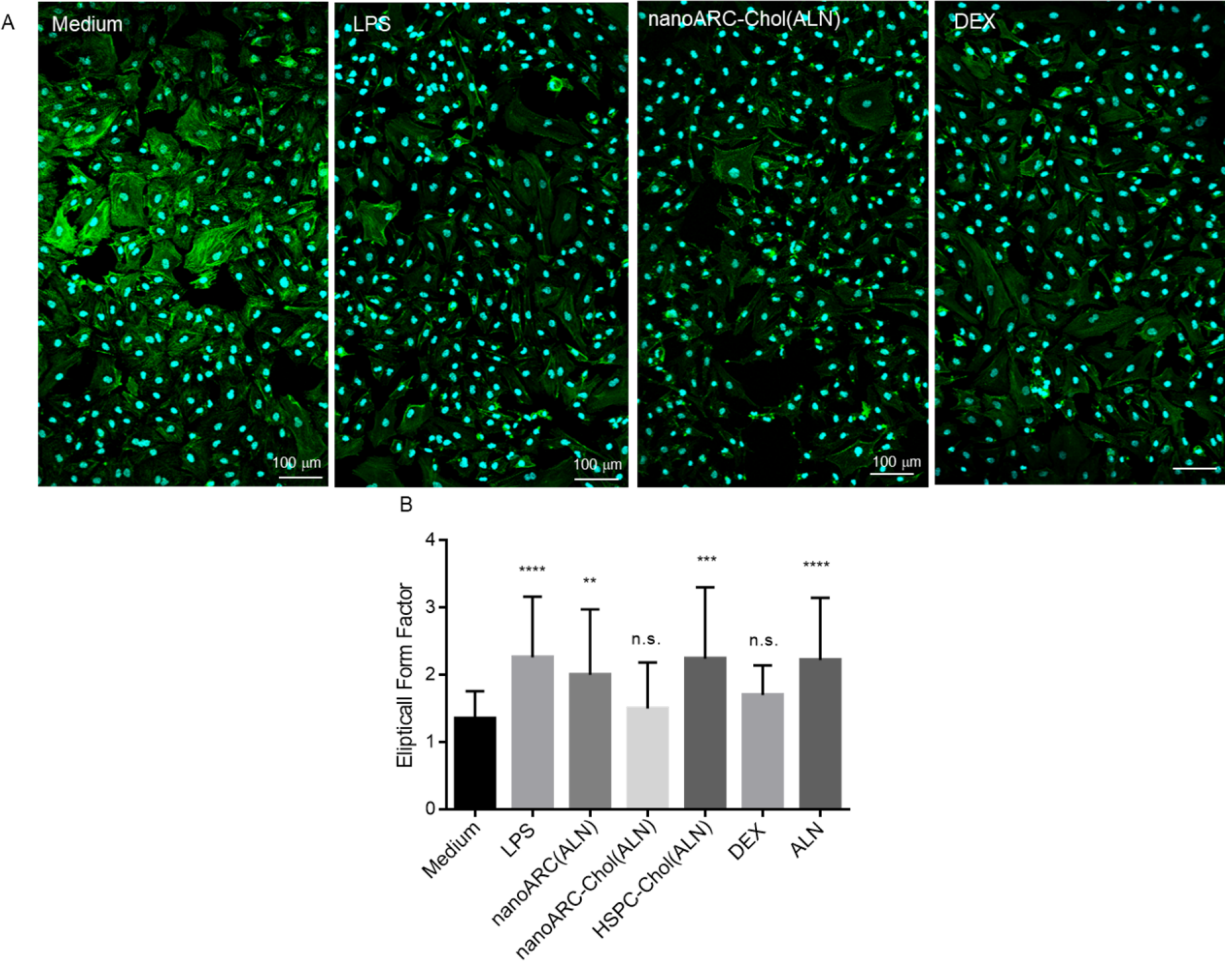
Morphological changes induced by LPS on HUVECs. (A) Representative fluorescence confocal microscopy images of HUVECs from the mild inflammation model. Cells were labelled with CytoPainter Phalloidin-iFluor 488 (actin) and Hoechst 33342 (nucleus). Scale bar: 100 µm. (B) Elliptical form factor (EFF). The EFF was calculated using ImageJ software analysis of cell morphology by dividing the major axis by the minor axis as an average of 100 cells [58].

A massive portion of endothelial cells is resistant to LPS challenge, entering a fibrotic program and exhibiting a fibroblast-like morphology through a process known as endothelial-to-mesenchymal transition (EndMT) typical from embryogenesis [53]. EndMT is observed under inflammatory conditions similar to the environment generated during sepsis or pathological processes such as renal, cardiac and pulmonary fibrosis, and cancer [54–57]. In the mild inflation model, LPS induced in HUVECs morphological changes compatible with EndMT, that is, a predominance of tapered cells with little internal staining. These changes were not prevented by almost any treatment, except dexamethasone and nanoARC-Chol (ALN), which maintained the endothelial morphology and the intensity of actin filament staining to a higher extent and cell/field ratio (Figure 12). Dexamethasone partly reduced the morphological changes induced on HUVECs but did not affect the IL-6/IL-8 production. In the pronounced inflammation model, the morphological changes experienced by HUVECs were not reversed by any treatment (data not shown).

## Discussion

The in vitro anti-inflammatory effects of ALN only occur at micromolar concentrations [12]. Such high concentrations, however, may hardly be achieved in blood because of the low bioavailability and high affinity of ALN to the hydroxyapatite matrix. At high oral dosages, ALN causes severe irritation of the upper gastrointestinal tract, osteonecrosis of the jaws (BRONJ), and serious musculoskeletal pain and cardiovascular risks [13,59–60]. Its intravenous delivery poses the risk of nephrotoxicity, fever, flu symptoms, and electrolyte imbalance [61]. Together, these disadvantages complicate its exploitation as an anti-inflammatory agent on cells other than osteoclasts or calcified vasculature. Formulated in nanoarchaeosomes, however, ALN could extend its effectiveness beyond an anti-osteoclast and anti-vascular calcifying agent. It could become an anti-inflammatory agent for HUVECs, more effective than dexamethasone.

Recent findings show that the co-culture of HUVECs with macrophages decreases the expression of eNOS [62], making them more likely to produce pro-inflammatory cytokines such as IL-6 and IL-8 and to express cell adhesion molecules, that is, making them more activated and pro-atherogenic [63]. Overall, the results of this research suggest that the endocytosis of one specific formulation, nanoARC-Chol(ALN), by irritated HUVECs in the mild inflammation model, was beneficial. The same as dexamethasone, nanoARC-Chol(ALN) was partly capable of reducing the morphological changes caused by LPS, potentially caused by tyrosine phosphorylation, actin depolymerization, and gap formation on the actin cytoskeleton [64]. However, the most important finding reported here is that nanoARC-Chol(ALN) was the only formulation capable of reducing the secretion of IL-6 and IL-8 by HUVECs, not only in the mild but also in the pronounced inflammation model.

There is a lack of agents able to reduce the secretion of IL-6 and IL-8 by the activated endothelium, required to treat atheromatous microenvironments; in there, besides inducing dramatic barrier alterations, IL-6 constitute mitogenic stimuli for smooth muscle cells [65–66]. The direct deactivation and protection of the endothelium are also poorly considered in the current management of sepsis [67]. Dexamethasone, for instance, is a powerful anti-edema agent used in the treatment of brain tumors [68] and to reduce edema associated with sepsis [69]. Dexamethasone displays the greatest glucocorticoid potency and anti-inflammatory activity among corticosteroids and has even higher anti-inflammatory activity than non-steroidal anti-inflammatory drugs [70]. Surprisingly however, dexamethasone and highly concentrated free ALN (2.5 μg/mL: 7.6 μM) and HSPC-Chol(ALN) liposomes were unable to reduce the secretion of IL-6 and IL-8 by HUVECs. In contrast, the endocytosis of nanoARC-Chol(ALN) did reduce the secretion by nearly 75% and 65%, respectively in the mild, and by 60% and 40%, respectively, in the pronounced inflammation model (the latter in the presence of TNF-α) [71]. These results suggest that the anti-inflammatory effect of nanoARC-Chol(ALN) was mediated by the synergic synchronous intracellular delivery of both archaeolipids and ALN. ALN is an anti-inflammatory molecule involved in the production of endothelial NO [12], and nanoarchaeosomes are internalized through SRAI/II, a polyanion receptor, which may induce anti-inflammatory responses [72]. Alternatively, the more extensive internalization of nanoarchaeosomes could simply mediate a higher intracellular delivery of ALN. The absence of anti-inflammatory effects provided by HSPC-Chol(ALN), hence, would be due to the lower ALN internalization, resulting from the lower ALN/lipid ratio and lower endocytosis of the formulation. In either case, the mechanism of reduction of IL-6/IL-8 of ALN-loaded nanoarchaeosomes would differ from that mediated by the corticosteroid receptor since the corticosteroid dexamethasone did not affect HUVECs. The nanovesicles are internalized by endocytosis, a mechanism independent of their concentration and dose. Nanoarchaeosomes from *H. tebenquichense* are massively endocytosed, total or partly via SRA1, the receptor responsible for massive, high-rate internalization of negatively charged molecules and microorganisms [40,73]. Therefore, the endocytosis of ALN-loaded nanovesicles is expected to provide a massive intracellular delivery of ALN. A massive intracellular delivery of ALN, in turn, was expected to increase intracellular NO levels, reported to cause a strong anti-inflammatory effect on the endothelium [12]. However, we did not measure a significant increase in NO levels after nanoARC-Chol(ALN) endocytosis. Finding the reasons for the strong anti-inflammatory action of nanoARC-Chol(ALN), which was higher than that of nanoARC(ALN) (made of pure archaeolipids), will require additional biochemical studies. The long-term effect of the presence of archaeolipids (lipids from distant phylogenetic origin) on the membrane of viable cells is another topic deserving further deeper exploration.

## Conclusion

This is the first report showing that in an environment of intense irritation caused by LPS, the endocytosis of nanoARC-Chol(ALN) by HUVECs magnifies the anti-inflammatory activity of ALN, not only surpassing that of its free form but also that of dexamethasone.

## Experimental

### Reagents and materials

Hydrogenated soy phosphatidylcholine (HSPC) was purchased from Northern Lipids Inc. (BC, Canada). Alendronate sodium trihydrate was a gift of Gador S.A. (BA, Argentina). Cholesterol (Chol) was purchased from ICN Biomedicals Inc. Oil Red O (ORO), 6-dodecanoyl-*N*,*N*-dimethyl-2-naphthylamine (Laurdan), 3-(4,5-dimethylthiazol-2-yl)-2,5-diphenyltetrazolium bromide (MTT), matrix metalloproteases substrate (FS-6), Sephadex G-50, lipopolysaccharides from *Escherichia coli* 0111:B4 (LPS), Mitochondria Staining Kit (JC-1 dye), valinomycin, sodium dodecyl sulfate (SDS), dexamethasone (DEX), ammonium persulfate, phorbol 12-myristate 13-acetate (PMA), gelatin from bovine skin type B, and BSA-fraction V were obtained from Sigma-Aldrich (MO, USA). Lissamine™ rhodamine B 1,2-dihexadecanoyl-*sn*-glycero-3-phosphoethanolamine triethylammonium salt (RhPE), Hoechst 33342, Pierce™ BCA Protein Assay Kit, and CM-H2DCFDA (general oxidative stress indicator) were purchased from Thermo Fisher Scientific (MA, USA). CytoPainter Phalloidin-iFluor 488 was purchased from Abcam plc. (UK). Endothelial Growth Medium-2 (EGM-2) was obtained from Lonza (Swiss). Roswell Park Memorial Institute 1640 (RPMI), penicillin–streptomycin, glutamine, sodium pyruvate, and trypsin/ethylenediamine tetraacetic acid were purchased from Gibco^®^ by Life Technologies (NY, USA). Fetal bovine serum (FBS) was purchased from Internegocios S. A. (BA, Argentina). The other reagents were of analytic grade and purchased from Anedra, Research AG (BA, Argentina).

### Archaea growth and lipid extraction

The hyperhalophilic archaea *Halorubrum tebenquichense* was grown in basal medium supplemented with yeast extract and glucose in a 25 L custom-made stainless steel bioreactor at 40 °C and harvested after 72 h growth. Total polar archaeolipids (TPA) were extracted from biomass using the Bligh and Dyer method modified for extreme halophiles [74]. Around 700 mg TPA was isolated from each culture batch. The reproducibility of each TPA extract composition was routinely screened by phosphate content [75] and electrospray-ionization mass spectrometry [76] and ordered according to decrescent abundance as archaeol analog methyl ester of phosphatidylglycerophosphate (PGP-Me), archaeol analog phosphatidylglycerol (PG), (1-*O*-[α-ᴅ-mannose-(2′-SO_3_H)-(1′→2′)-α-ᴅ-glucose]-2,3-di-*O*-phytanyl-*sn*-glycerol) (S-DGD-5), the cardiolipin bisphosphatidylglycerol (BPG), and the glycocardiolipin (2′-SO_3_H)-Manp-α-1,2-Glcpa-1–1-[*sn*-2,3-di-*O*-phytanylglycerol]-6-[phospho-*sn*-2,3-di-*O*-phytanylglycerol] (SDGD-5PA) (Figure 1).

### Preparation and characterization of nanovesicles

In a manner analogous to [29], nanoarchaeosomes made of TPA (nanoARC) and of TPA/Chol 7:3 w/w (nanoARC-Chol), and nanoliposomes made of HSPC/Chol 7.5:2.5 w/w, were prepared by the film hydration method. To that aim, blends of lipids were dissolved in chloroform/methanol 1:1 v/v, and the solvents were rotary-evaporated until complete removal. The lipid films were flushed with N_2_ and hydrated with Tris buffer (10 mM Tris-HCl buffer pH 7.4 with NaCl 0.9% w/w) to obtain the void nanovesicles or 14 mg/mL ALN in Tris buffer (to obtain ALN-containing nanovesicles) up to a final concentration of 10 mg/mL total lipids at room temperature for nanoarchaeosomes and 60 °C for HSPC-Chol. The suspensions of nanovesicles were sonicated for 1 h employing a bath-type sonicator (80 W, 80 kHz) and extruded 10–15 times through 0.4 μm and 0.2 μm pore size polycarbonate filters using a thermobarrel extruder (Northern Lipids, Inc. BC, Canada).

Free ALN was removed by gel filtration on Sephadex G-50. Briefly, aliquots of 300 μL of nanovesicles were poured on a 3 mL syringe packed with Sephadex G-50, centrifuged for 5 min at 700*g*, and the first fraction of 250–300 μL was collected. The resultant nanovesicles were sterilized by passage through a 0.22 μm sterile filter and stored at 4 °C.

ALN was quantified in the aqueous phase, while phospholipids were quantified in the chloroform phase after extraction of ALN using the Bligh and Dyer method [77] by a colorimetric phosphate microassay [75].

Size and ζ potential of nanovesicles were determined by dynamic light scattering and phase analysis light scattering, respectively, using a Zetasizer Nano ZS apparatus (Malvern Instruments Ltd, UK).

To prepare RhPE-labeled nanovesicles, RhPE at 0.4 μg per mg of lipids was added to the mixture of lipids, and lipid films were hydrated with Tris buffer as stated above. RhPE was quantified by spectrofluorometry (λ_ex_ = 561 nm and λ_em_ = 580 nm) with an LS55 fluorescence spectrometer (PerkinElmer Inc. MA, USA) upon complete disruption of 1 volume of nanovesicles in 10 volumes of methanol. The fluorescence intensity of the sample was compared with a standard curve prepared with RhPE in methanol. The standard curve was linear in the range of 0.075–0.5 μg/mL RhPE.

### Cells and culture conditions

Human umbilical vein endothelial cells (HUVECs) were obtained by Dr. Nancy Lorena Charó from the Institute of Experimental Medicine, CONICET-National Academy of Medicine, with the tenets of the Declaration of Helsinki and a protocol previously approved by the institution’s ethics committee. Briefly, the umbilical cord was collected after normal deliveries with written informed consent from the mother. HUVECs were purified from the human umbilical vein by digestion with collagenase (Gibco, Grand Island, NY, USA) [78]. Cells were grown in plate coated with 2% gelatin from bovine skin type B in EGM-2 supplemented with antibiotics (100 U/mL penicillin and 100 µg/mL streptomycin). HUVECs were used between the first and fourth passages.

The human monocyte cell line THP-1 was grown in RPMI medium supplemented with 10% FBS, 100 U/mL penicillin, 100 μg/mL streptomycin, 0.05 mM 2-mercaptoethanol, 1 mM sodium pyruvate, and 2 mM ʟ-glutamine in a humidified atmosphere of 5% CO_2_ at 37 °C. THP-1 cells were differentiated into macrophages by treatment with 25 nM of PMA in RPMI medium without 2-mercaptoethanol and pyruvate for 48 h following 24 h of incubation in RPMI medium [79].

Foam cells (FCs) were induced from THP-1 macrophages by incubation with oxidized LDL (ox-LDL) as described by Ledda and co-workers [80]. Briefly, 1.5 × 10^4^ THP-1 macrophages were incubated with 100 μg/mL of human oxLDL for 24 h at 37 °C and 5% CO_2_ atmosphere. FC induction was assessed by ORO staining. Briefly, cells were fixed to glass coverslips previously placed in 24-well plates by covering them with 10% formaldehyde in PBS for 15 min at room temperature (RT). After removing the fixing buffer carefully, cells were covered with fresh ORO working solution (6 mL of 5 mg/mL ORO in isopropanol stock solution) and 4 mL of distilled water filtered through a 3 μm pore size filter) for at least 1 h at RT. Then cells were rinsed several times carefully with distilled water and allowed to air-dry. Cells were visualized under an optical microscope Olympus BX51 equipped with an Oan Olympus DP-70 camera (Olympus, Japan). The designation of a macrophage as FC required positive ORO staining [81].

### Cytotoxicity

In a manner analogous to [29], the viability of THP-1 macrophages and HUVECs upon 24 h of incubation with free ALN, or void or ALN-loaded nanovesicles was determined by the MTT assay. To that aim, 2 × 10^4^ THP-1 macrophages or 5 × 10^3^ HUVECs per well were seeded into 96-well plates and grown for 24 h. Then, cells were incubated with 100 µL nanovesicles at 10, 50, 100, and 500 μg/mL total lipid (TL) or ALN. After 24 h of incubation, the medium was removed, cells were washed once with 100 µL PBS, and 100 μL of 0.5 mg/mL MTT solution was added to each well. After 3 h of incubation, the MTT solution was removed, the insoluble formazan crystals were dissolved in dimethyl sulfoxide (DMSO), and absorbance was measured at 570 nm in a Cytation™ 5 cell imaging multi-mode reader (Biotek Instruments, VT, USA). The cell viability was expressed as a percentage of the cells grown in the medium.

The viability of THP-1 monocytes upon 30 and 180 min of co-incubation with void and ALN-loaded nanovesicles and LPS was measured by the MTT assay modified for suspension cell lines [82]. Briefly, 4 × 10^4^ THP-1 cells in 50 μL of RPMI medium were seeded into 96-well plates and grown for 4 h. Then, 50 μL of nanovesicles or ALN dilutions in RPMI medium with 1 μg/mL LPS was added to each well to reach the final concentration of 100 and 500 μg/mL TL or 5 and 25 μg/mL ALN. After incubation, plates were centrifuged at 125*g* for 5 min, the medium was removed, and cells were washed once with 100 µL PBS and incubated for 24 h in RPMI medium with 5% SFB. Then 5 μL of 5 mg/mL MTT solution was added to each well. After 4 h of incubation, 90 μL of DMSO and 60 μL of SDS lysis solution (0.3 g/mL SDS pH 1.7) were added to each well and shaken in an orbital shaker at 120 rpm in the darkness for 15 min. Finally, the absorbance was measured at 550 nm as stated before.

### Uptake of nanovesicles

The uptake of RhPE-labelled nanovesicles by THP-1 monocytes and macrophages was measured by flow cytometry. Briefly, THP-1 monocytes were seeded on 24-well culture plates at a density of 1 × 10^6^ cells per well (150 µL), and THP-1 macrophages were seeded on 24-well culture plates at a density of 1.5 × 10^5^ cells per well and grown for 24 h. Then, the cells were incubated with 100 μg/mL TL of RhPE nanovesicles in complete medium for 1 and 5 h at 37 °C. After incubation, THP-1 macrophages were trypsinized. THP-1 monocytes and trypsinized macrophages were washed once with 300 µL PBS, and a total of 1 × 10^4^ cells were analyzed by flow cytometry (BD FACSCalibur™; BD Biosciences, San Jose, CA, USA). The fluorescence was normalized to the RhPE/TL ratio of each formulation. Data were analyzed using Flowing Software 2.5.1 (Flowing Software, Finland).

#### Plasma membrane order

Effects of nanovesicle uptake on THP-1 macrophages and HUVEC plasma membrane order were determined by measuring the Laurdan generalized polarization (GP) [83]. Briefly, THP-1 macrophages and HUVECs grown at a density of 1–1.5 × 10^4^ cells/cm^2^ on 96-well plates were incubated with 100 μg/mL TL (THP-1 macrophages) or 50 μg/mL TL (HUVECs) of nanovesicles for 24 h at 37 °C. After incubation, supernatants were discarded, and cells were washed once with 100 µL PBS and incubated with 5 μM of Laurdan for 1 h at 37 °C. Then, supernatants were discarded, cells were washed with PBS, and the fluorescence intensity was measured in each well with a Cytation™ 5 instrument using λ_ex_ = 350/10 nm, λ_em_ = 440/10 nm, and λ_em_ = 490/10 nm. GP was calculated using the following equation: GP = (I440 − I490)/(I440 + I490) where I440 and I490 are the fluorescence intensities at λ_em_ = 440 nm and λ_em_ = 490 nm, respectively.

### Mitochondrial membrane potential

The effect of nanovesicle uptake by THP-1 macrophages and HUVECs on the mitochondrial membrane potential was determined using JC-1 dye according to the manufacturer’s guidelines [84]. Briefly, THP-1 macrophages and HUVECs were seeded and incubated with nanovesicles as stated before. After incubation, supernatants were discarded, and cells were washed once with 100 µL PBS and incubated with 2.5 μg/mL JC-1 staining mixture for 15 min at 37 °C. Upon supernatant removal and PBS washing, the fluorescence intensity of each well was measured in a Cytation™ 5 instrument. The fluorescence of JC-1 monomers was determined at λ_ex_ = 490/10 nm and λ_em_ 530/20 nm, and that of JC-1 aggregates at λ_ex_ = 525/20 nm and λ_em_ = 590/20 nm. Positive control was carried out by incubating cells with 100 ng/mL valinomycin.

### Intracellular ATP content

The effect of nanovesicle uptake by THP-1 macrophages and HUVECs on the intracellular ATP content was determined with CellTiter-Glo^®^ luminescent cell viability assay (Promega, WI, USA) according to the manufacturer’s guidelines. Briefly, THP-1 macrophages and HUVECs were seeded and incubated with nanovesicles as stated before. After that, supernatants were discarded, and cells were washed once with 100 µL PBS and incubated with fresh medium for 30 min at RT. Then, one volume of CellTiter-Glo^®^ reactive was added to cell media in each well, stirred for 2 min in an orbital shaker and incubated for 10 min at RT until signal stabilization. The luminescence of each well was measured in a Cytation™ 5 instrument.

### Matrix metalloproteinases (MMP)

The release of MMP by THP-1 macrophages 24 h after nanovesicle uptake was determined by measuring the fluorescence of the peptide FS-6. The FS-6 peptide (MCA-Lys-Pro-Leu-Gly-Leu-DNP-Dpa-Ala-Arg-NH_2_) is a fluorogenic substrate with improved substrate properties and increased specific constant for collagenases (MMP-1, MMP-8, and MMP-13) and MT1-MMP (MMP-14) compared with FS-1 [84]. Briefly, THP-1 macrophages grown at a density of 2 × 10^4^ cells/cm^2^ on 96-well plates and were co-incubated with 100 or 50 μg/mL TL of nanovesicles, 2.5 µg/mL ALN, or 10 µg/mL Dex, and 1 µg/mL LPS in RPMI medium without phenol red, with heat-inactivated SFB for 24 h at 37 °C. After incubation, supernatants were collected and transferred to black 96-well plates, and FS-6 at 5 µM final concentration in the well was added and incubated for 6 h at 37 °C. Fluorescence intensity was measured each 30 min in a Cytation™ 5 instrument at λ_ex_ = 320/10 nm and λ_em_ = 405/10 nm. The activity of MMP was expressed as relative fluorescence units per minute [85].

### Vascular inflammation models: endothelial cell/macrophage co-cultures

#### Co-culture preparation

To explore the effect of ALN-loaded nanoarchaeosomes on irritated HUVECs, we prepared two inflammation models where the HUVECs were seeded on the apical side of a porous membrane that separated the upper from the basal compartment. The HUVECs were submitted to LPS either on the apical side (mild inflammation model) or on the apical and basal sides (pronounced inflammation model); to increase the irritation caused by LPS, the former contained THP1-derived macrophages and the latter FCs grown on the basal side. Both cell types respond to LPS stimulation with more irritation; FCs are known to facilitate pathogenesis by producing eicosanoids, tissue-damaging enzymes, and extracellular vesicles [86]. The resultant responses diffused either downwards or upwards across the porous membrane, constituting what we called a synchronous, conditioned medium.

**Figure 13:**
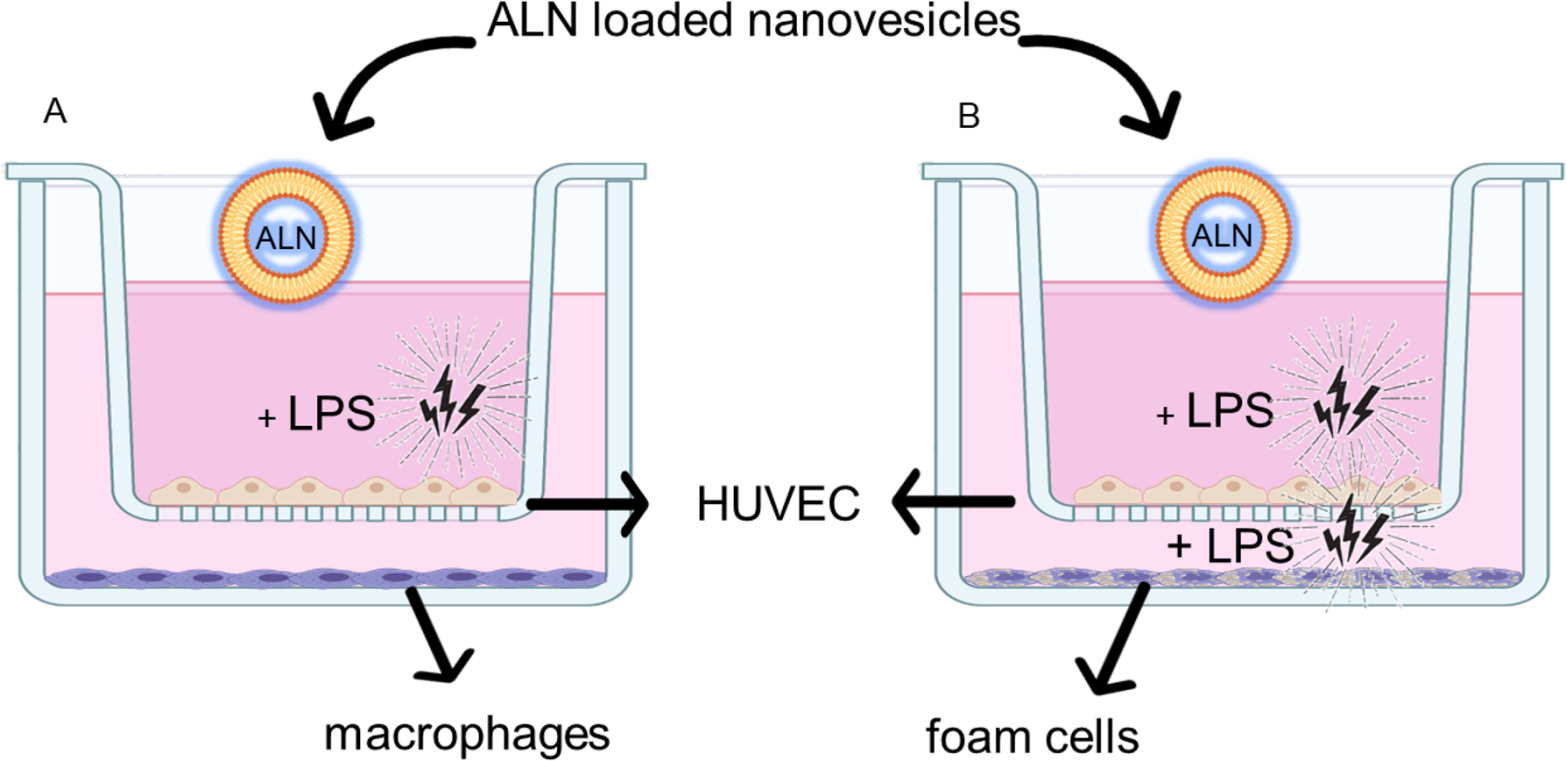
(A) Mild inflammation model. (B) Pronounced inflammation model.

Before being irritated with LPS, HUVECs were incubated with macrophages or FCs for 4 h to engage in a crosstalk that took the HUVECs to a more pathophysiological relevant microenvironment, characterized by the secretion of IL-6 and IL-8. A scheme of the two models is shown in Figure 13.

Briefly, HUVECs were seeded in EGM-2 medium at a density of 4 × 10^4^/cm^2^ on ThinCert™ cell culture inserts (12 wells, 0.4 µm pore size PET membrane Greiner Bio-One GmbH, Austria) previously covered with 2% bovine skin gelatin type B and grown for 24 h. Also, 10.5 × 10^4^/cm^2^ THP-1 macrophages per well were seeded on a different 12-well culture plate. Then, the inserts with HUVECs were transferred to the plates with differentiated THP-1 macrophages. A crosstalk between both cell types was established by incubating for 24 h. Then inflammation was induced by incubation with 1 μg/mL of LPS on the upper compartment.

For the pronounced inflammation model, THP-1 macrophages were transformed into FCs as stated before. Then, the inserts with HUVECs were transferred to the plates with FCs, and a crosstalk was established by incubating for 24 h. Then, inflammation was induced by incubation with 1 μg/mL of LPS on the upper and the basolateral compartments.

#### Anti-inflammatory and antioxidant activity on co-cultures

Anti-inflammatory and antioxidant activity of nanovesicles on mild and pronounced inflammation models were determined by measuring the production of IL-8, IL-6, and TNF-α in the apical and basolateral compartments, and the ROS production on differentiated THP-1 cells and FCs. Briefly, ALN-loaded nanovesicles at 50 μg/mL TL + 2.5 μg/mL ALN, free ALN at 2.5 μg/mL, or DEX 10 μg/mL were applied on the apical compartment, and LPS was added at 1 μg/mL. Upon 18 h of incubation at 37 °C, supernatants of upper and basolateral compartments were collected and stored at −20 °C for further analysis. Human TNF-α, IL-8, and IL-6 levels were measured by enzyme-linked immunosorbent assay (BD OptEIA™, BD Biosciences, San Jose, CA, USA) following the manufacturer’s guidelines. Absorbance measurements were carried out at 450 nm on a microplate reader.

The generation of ROSs was measured in THP-1 macrophages and FCs in the basolateral compartments using the CM-H2DCFDA dye. Briefly, cells attached at the bottom of the well were washed twice with 100 µL PBS and incubated with a solution of 10 µM CM-H2DCFDA for 30 min at 37 °C. Then, cells were washed with PBS, and the fluorescence intensity of whole cells was measured using a Cytation™ 5 instrument at λ_ex_ = 490 nm and λ_em_ = 520 nm.

Confocal microscopy was performed to study the morphology of HUVECs. Briefly, after incubation, cell monolayers were washed with PBS and fixed with 3.7% formaldehyde in PBS for 30 min. Then, cells were permeabilized with 0.1% Triton X-100 in PBS for 10 min, followed by 30 min incubation with 1% bovine serum albumin (BSA) in PBS. Finally, cells were incubated with CytoPainter Phalloidin-iFluor 488 [87] for 90 min and Hoechst 33342 for 10 min at RT. After staining, the membranes were separated from the inserts and were mounted on slides using a motion mounting medium. A Leica laser-scanning spectral confocal microscope TCS SP8 (Leica Microsystems, Wetzlar, Germany) was used. Image processing was performed using ImageJ software (National Institutes of Health).

#### NO production

The nitric oxide production in HUVECs was measured by the determination of nitrite, a stable and non-volatile breakdown product of the NO released, in the incubation medium employing the spectrometric Griess reaction [12]. Briefly, cells were seeded on 24-well culture plates at a density of 3.5 × 10^4^ cells/well in EGM-2 medium containing 10% (v/v) FBS. Then, cells were incubated with 50 μg/mL TL of ALN-loaded nanovesicles or 1, 5, 10, and 50 µM ALN in EGM-2 medium containing 1% (v/v) FBS for 15, 30, and 60 min at 37 °C. Once the treatment was finished, aliquots of culture medium supernatant were mixed with 1 volume of Griess Reactive Solution “A” and 1 volume of Griess Reactive Solution “B” and incubated 15 min at room temperature protected from the light. Absorbance was measured at 520 nm in a Cytation™ 5 instrument. Nitrite concentration in the samples was determined with reference to a sodium nitrite (NaNO_2_) standard curve performed in the same medium from 10 to 50 µM.

### Statistical analysis

Statistical analyses were performed by one-way analysis of variance followed by Dunnet’s test or two-way analysis of variance followed by Sildak’s test using Prisma 4.0 Software (Graph Pad Software, CA, USA). A *p*-value < 0.05 was considered statistically significant: *p < 0.05; **p < 0.01; ***p < 0.001, ****p < 0.0001; n.s. represents non-significant (*p* > 0.05).

## Supporting Information

File 1 Additional figures.

## Data Availability

All data that supports the findings of this study is available in the published article and/or the supporting information to this article.
