# Supplementary material for: Cholesterol nanoarchaeosomes for alendronate targeted delivery as an anti-endothelial dysfunction agent
Source: Beilstein J Nanotechnol. 2024 May 13;15:517–34. doi: 10.3762/bjnano.15.46 (PMC11106671; doi:10.3762/bjnano.15.46)
Supplement: File 1 — Additional figures. [file Beilstein_J_Nanotechnol-15-517-s001.pdf]

BEILSTEIN JOURNAL OF NANOTECHNOLOGY

Supporting Information

for

Cholesterol nanoarchaeosomes for alendronate targeted delivery as an anti-endothelial dysfunction agent

Horacio Emanuel Jerez, Yamila Roxana Simioni, Kajal Ghosal, Maria Jose Morilla and Eder Lilia Romero

Beilstein J. Nanotechnol. **2024,** *15,* 517–534. doi:10.3762/bjnano.15.46

Additional figures

License and Terms: This is a supporting information file under the terms of the Creative Commons Attribution License (https://creativecommons.org/licenses/by/4.0). Please note that the reuse, redistribution and reproduction in particular requires that the author(s) and source are credited and that individual graphics may be subject to special legal provisions.
The license is subject to the *Beilstein Journal of Nanotechnology* terms and conditions: (https://www.beilstein-journals.org/bjnano/terms)

A

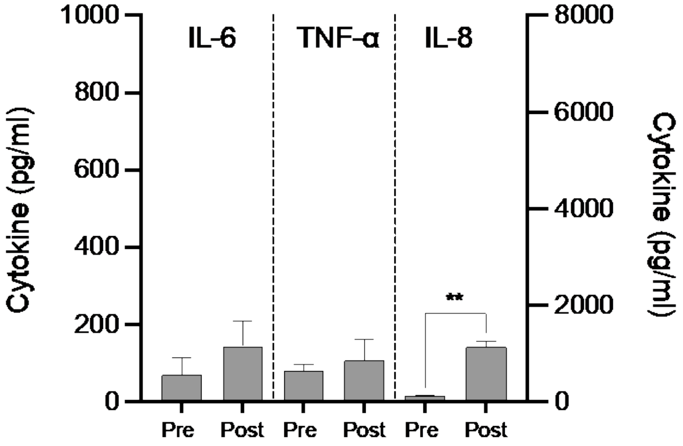

B

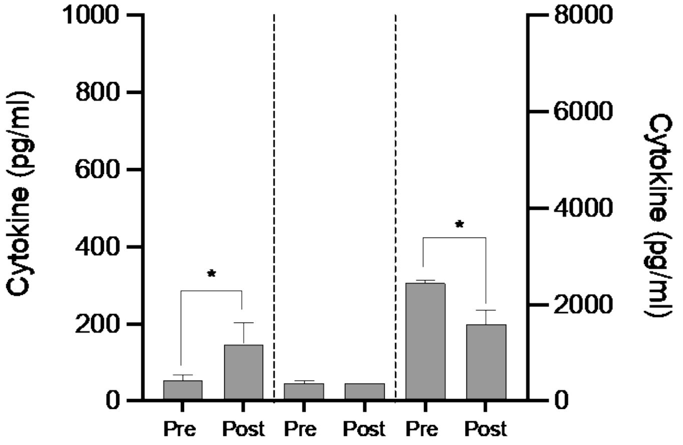

Figure S1: Cytokine release in the compartments of the mild inflammation model before (pre) and after (post) 24 h of crosstalk incubation: (A) apical (HUVECs) and (B) basolateral (THP-1 macrophages). Values of IL-6 and TNF-α are shown on the left *Y* axis, and values of IL-8 are shown on the right *Y* axis. Data are expressed as mean ± SD (*n* = 2).

A

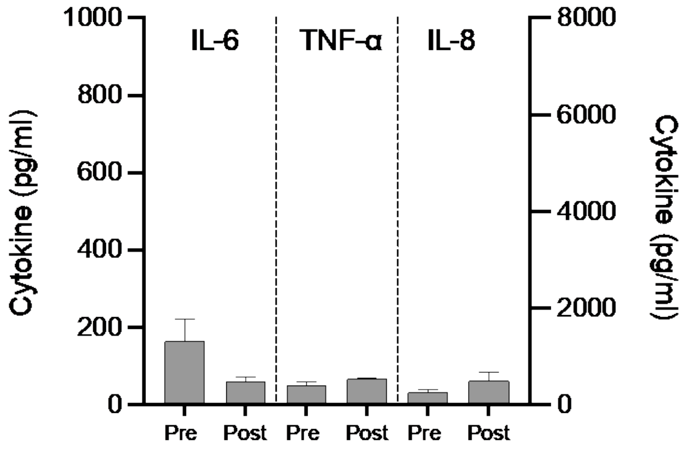

B

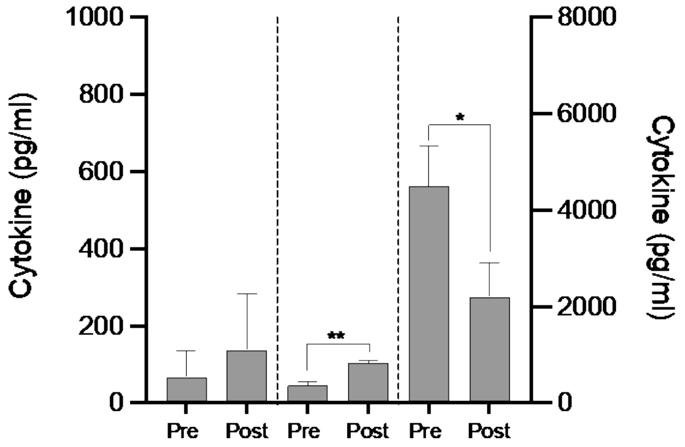

Figure S2: Cytokine release in the compartments of the pronounced inflammation model before (pre) and after (post) 24 h of crosstalk incubation: (A) apical (HUVECs) and (B) basolateral (FCs). Values of IL-6 and TNF-α are shown on the left *Y* axis, and values of IL-8 are shown on the right *Y* axis. Data are expressed as mean ± SD (n = 2).

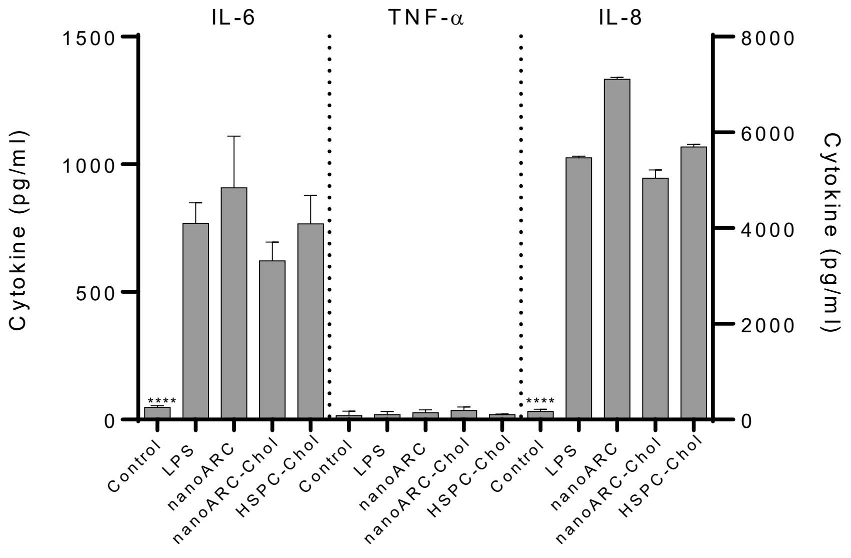

Figure S3: Cytokine release in the upper compartment of the mild inflammation model after incubation with void vesicles. Values of IL-6 and TNF-α are shown on the left *Y* axis, and values of IL-8 are shown on the right *Y* axis. Data are expressed as mean ± SD (*n* = 2).
